# STAT3 Is the Master Regulator for the Forming of 3D Spheroids of 3T3-L1 Preadipocytes

**DOI:** 10.3390/cells11020300

**Published:** 2022-01-16

**Authors:** Hiroshi Ohguro, Yosuke Ida, Fumihito Hikage, Araya Umetsu, Hanae Ichioka, Megumi Watanabe, Masato Furuhashi

**Affiliations:** 1 Departments of Ophthalmology, School of Medicine, Sapporo Medical University, Sapporo 060-8556, Japan; ooguro@sapmed.ac.jp (H.O.); funky.sonic@gmail.com (Y.I.); fuhika@gmail.com (F.H.); araya.alaya.favreweissth@gmail.com (A.U.); ichioka29@sapmed.ac.jp (H.I.); watanabe@sapmed.ac.jp (M.W.); 2 Departments of Cardiovascular, Renal and Metabolic Medicine, School of Medicine, Sapporo Medical University, Sapporo 060-8556, Japan

**Keywords:** 3D spheroid culture, 3T3-L1 preadipocyte, RNA sequencing, Gene Ontology (GO) enrichment analysis, Ingenuity Pathway Analysis (IPA)

## Abstract

To elucidate the currently unknown mechanisms responsible for the diverse biological aspects between two-dimensional (2D) and three-dimensional (3D) cultured 3T3-L1 preadipocytes, RNA-sequencing analyses were performed. During a 7-day culture period, 2D- and 3D-cultured 3T3-L1 cells were subjected to lipid staining by BODIPY, qPCR for adipogenesis related genes, including *peroxisome proliferator-activated receptor γ* (*Pparγ*), *CCAAT/enhancer-binding protein alpha* (*Cebpa*), *Ap2* (*fatty acid-binding protein 4; Fabp4*), *leptin*, and *AdipoQ* (*adiponectin*), and RNA-sequencing analysis. Differentially expressed genes (DEGs) were detected by next-generation RNA sequencing (RNA-seq) and validated by a quantitative reverse transcription–polymerase chain reaction (qRT–PCR). Bioinformatic analyses were performed on DEGs using a Gene Ontology (GO) enrichment analysis and an Ingenuity Pathway Analysis (IPA). Significant spontaneous adipogenesis was observed in 3D 3T3-L1 spheroids, but not in 2D-cultured cells. The mRNA expression of *Pparγ*, *Cebpa,* and *Ap2* among the five genes tested were significantly higher in 3D spheroids than in 2D-cultured cells, thus providing support for this conclusion. RNA analysis demonstrated that a total of 826 upregulated and 725 downregulated genes were identified as DEGs. GO enrichment analysis and IPA found 50 possible upstream regulators, and among these, 6 regulators—transforming growth factor β1 (TGFβ1), signal transducer and activator of transcription 3 (STAT3), interleukin 6 (IL6), angiotensinogen (AGT), FOS, and MYC—were, in fact, significantly upregulated. Further analyses of these regulators by causal networks of the top 14 predicted diseases and functions networks (IPA network score indicated more than 30), suggesting that STAT3 was the most critical upstream regulator. The findings presented herein suggest that STAT3 has a critical role in regulating the unique biological properties of 3D spheroids that are produced from 3T3-L1 preadipocytes.

## 1. Introduction

The number and sizes of adipocytes, which are elastic and plastic cells in fat tissue, are altered during adipogenic differentiation or are eliminated through necrosis or apoptosis in response to fluctuations in metabolism states [1,2,3]. Since those characteristic changes in adipocytes occur within the three-dimensional (3D) spaces of the body, a 3D cell culture method would be expected to be more representative than conventional two-dimensional (2D) cell culture methods in adipocyte-related research. However, at the time of writing this article, only limited studies have been conducted using a 3D cell culture method due to the difficulties associated with these techniques, even though 3D tissue cultures have emerged as a useful tool for modeling several human diseases [4]. In fact, this 3D culture technique has the potential for developing an understanding of spatial cell–cell and cell–extracellular-matrix (ECM) interactions that could not be possible in conventional 2D culture models [5]. In a recent report, we reported on the production of 3D spheroids of 3T3-L1 cells and human orbital adipocytes by a 3D drop culture technique [6] and, using those, we were able to establish in vitro models that replicate prostaglandin-induced orbital fat atrophy, a condition called “deepening of the upper eyelid sulcus (DUES)” [7], and thyroid-associated orbitopathy [6]. During these studies, we identified some rather interesting and unidentified differences, in that the 3D spheroids showed some distinct differences from the conventional 2D cultures. Included among these differences were that (1) 2D 3T3-L1 cells were rapidly dispersed within a few minutes in the presence of 0.2% trypsin, whereas 3D 3T3-L1 preadipocyte spheroids started to disperse only after 3 h, and up to 12 h was needed for this process to reach completion, and 3D 3T3-L1 adipocytes were relatively resistant to 0.2% trypsin for periods of up to 12 h, and (2) adipogenic differentiation was much more efficient in the case of 3D 3T3-L1 spheroids, compared with 2D-cultured 3T3-L1 cells [8,9,10,11,12]. Therefore, this collective body of evidence suggested that 3D spheroids may be more biologically active than 2D cultures, even though the 3T3-L1 cells were cultured under exactly the same conditions, except that one group was 2D and the other 3D. Furthermore, no rational explanation has been available that explains why round-shaped 3D spheroids are formed within the teardrop composed of a cell culture medium.

Therefore, in the present study, to elucidate the above unknown mechanisms for causing such a distinct phenomenon, we carried out an RNA-sequencing analysis, which is a powerful tool for identifying potential target genes for diseases and the underlying pathological mechanisms [13], between 2D- and 3D-cultured 3T3-L1 cells.

## 2. Materials and Methods

### 2.1. Two-Dimensional (2D) and Three-Dimensional (3D) Cultures of 3T3-L1 Cells

The 3T3-L1 preadipocytes (#EC86052701-G0, KAK), a cell line that is universally used in lipid studies, were cultured in 2D culture dishes at 37 °C in a grown medium; high glucose Dulbecco’s modified Eagle medium (HG-DMEM, FUJIFILM, Osaka, Japan) supplemented with 8 mg/L d-biotin, 4 mg/L calcium pantothenate, 100 U/mL penicillin, 100 μg/mL streptomycin (b.p. HG-DMEM), 10% CS and methylcellulose (Methocel A4M, Sigma-Aldrich, St. Louis, MO, USA) as a morphology stabilizer.

To generate 3D spheroids under culture conditions identical to those used for the 2D cell culture except culture plates, a hanging droplet spheroid 3D culture system, described recently [6,14], was used. Briefly, 3T3-L1 cells were cultured in 100 mm or 150 mm dishes as described above. After reaching approximately 90% confluence, the cells were detached by treatment with 0.25% trypsin/EDTA after washing with phosphate-buffered saline (PBS) and then resuspended in the growth medium. These cell suspensions were divided into conventional 2D cultures and 3D spheroid cultures. The 2D-cultured 3T3-L1 cells were again maintained 100 mm 2D culture dishes until Day 7, with medium changes daily. Alternatively, a cell suspension containing approximately 20,000 cells in a 28 μL culture medium was then placed into each well of the 3D drop culture plate (# HDP1385, Sigma-Aldrich, St. Louis, MO, USA). This timing was defined as 3D/Day 0, and 14 μL of the culture medium was then replaced with 14 μL of fresh culture medium in each well daily until Day 7 [7,8,10]. On Day 7, both 2D- and 3D-cultured 3T3-L1 cells were each collected and further processed for use in the RNA-sequencing analysis, as described below.

### 2.2. Lipid Staining by BODIPY

Lipid staining with BODIPY was performed as described previously [8,9,10]. Briefly, 2D or 3D 3T3-L1 cells were incubated in a mixture of 0.2% BODIPY (# D3922, Thermo Fisher Scientific, Waltham, MA, USA), Alexa Fluor 594 phalloidin (# 20553, Funakoshi, Tokyo, Japan), and DAPI (# D523, Dojindo, Kumamoto, Japan), at 1:1000 dilutions in PBS for 1 hr, and thereafter fixed in 4% paraformaldehyde in PBS for 10 min at room temperature. Fluorescence intensities were measured using a Nikon A1 confocal microscope (Tokyo, Japan) and quantified using Image J software version 2.0.0 (NIH, Bethesda, MD, USA).

### 2.3. RNA-Sequencing Analyses

Isolation of total RNA from 2D confluent cells of 150 mm dish or 3D 3T3-L1 spheroids (*n* = 80), prepared as described above, were performed in duplicate, using an RNeasy mini kit (QIAGEN, Valencia, CA, USA) according to the manufacturer’s instructions; the procedure further processed as follows: RNA content and quality were measured using NanoPhotometer^®^ P330 (IMPLEN, Los Angeles, CA, USA) and an Agilent 2100 Bioanalyzer (Agilent Technologies, Massy, France), respectively. As the quality of the RNA was suitable for RNA sequencing and quantitative real-time PCR, the samples with an RNA integrity number (RIN) > 8.5 were confirmed in advance. cDNA libraries were prepared using a TruSeq RNA Sample Preparation Kit (Illumina, San Diego, CA, USA) [15], and their quality and quantity were determined using an Agilent 2100 Bioanalyzer and KAPA Library Quantification Kit (KAPA Biosystems, Wilmington, MA, USA) [16], respectively. Cluster generation, sequencing the multiplexed samples, image analysis, base calling, and quality filtering were performed as described in a previous report (Illumina, San Diego, CA, USA). Sequence data were filtered by the removal of the adapter sequence, ambiguous nucleotides, and low-quality sequences. The remaining sequence data were then aligned according to the Swine genome sequence (susScr3). The expression values for each respective gene and statistical analysis of the differentially expressed genes were expressed using the mapping of sequence data (QIAGEN, Redwood City, CA, USA). Statistical significance was determined by the empirical analysis. Using a moderated *t*-test with the *p* value corrected using the Benjamini–Hochberg algorithm, genes with fold change ≧2.0 and FDR-adjusted *p* < 0.05 and *q* < 0.2 were assigned as differentially expressed genes (DEG).

### 2.4. Gene Function and Pathways Analyses

For estimating gene function, a Gene Ontology (GO) enrichment analysis was performed as described in a previous study [17]. During the GO enrichment analysis, molecular function, biological processes, cellular components, etc. with a *p* value less than 0.05 were considered to be significantly enriched by differentially expressed genes based upon the Kyoto Encyclopedia of Genes and Genomes (KEGGs), a database resource for understanding high-level functions and the effects of the biological system (http://www.genome.jp/kegg/, accessed on 3 November 2021).

To predict possible upstream transcriptional regulators, DEGs were interpreted using the upstream regulator function of the Ingenuity Pathway Analysis (IPA, QIAGEN, https://www.qiagenbioinformatics.com/products/ingenuity-pathway-analysis, accessed on 3 November 2021). As a predicted function, pathway, or upstream regulator (activation or deactivation), the activation z-score algorithm was used, in which the number of standard deviations in the data lies above or below the mean. A z-score ≥2 was considered to be significantly increased, whereas a z-score ≤−2 was considered significantly decreased [18].

### 2.5. Quantitative PCR

Total RNA extraction (RNeasy Mini Kit, QIAGEN, Valencia, CA, USA), reverse transcription (SuperScript IV Kit, Invitrogen, Waltham, MA, USA), and real-time PCR (Applied Biosystems/Thermo Fisher Scientific, Waltham, MA, USA) were performed as described previously [7,8,10]. The housekeeping 36B4 (*Rplp0*) gene was used for the normalization of the cDNA levels of the respective genes. The sequences of the primers and Taqman probes used are shown in Supplementary Table S1.

### 2.6. Statistical Analysis

All statistical analyses were performed using GraphPad Prism 8 (GraphPad Software, San Diego, CA, USA). To analyze the difference between groups, an unpaired *t*-test was performed. Data are expressed as the arithmetic mean ± the standard error of the mean (SEM).

## 3. Results

### 3.1. Spontaneous Sdipogenesis in 3D 3T3-L1 Spheroids

**Figure 1:**
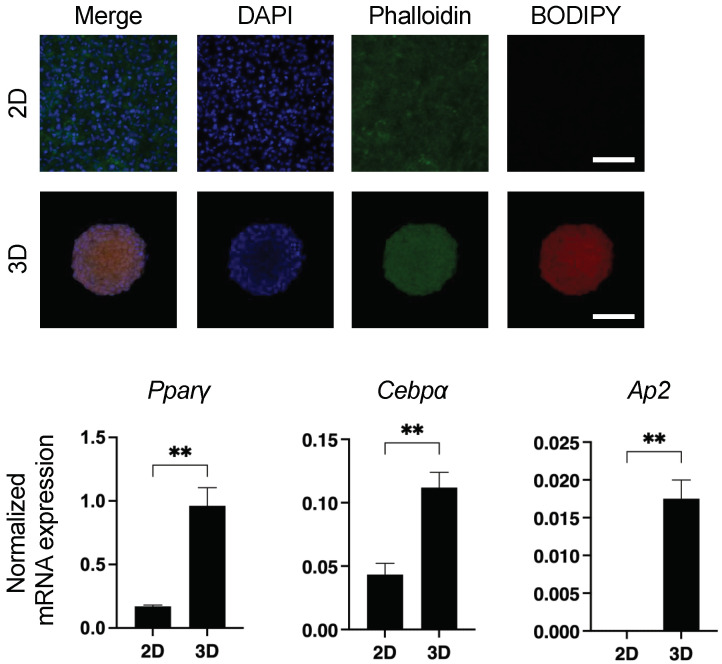
Spontaneous adipogenic differentiation in 3D 3T3-L1 spheroids. Representative images of lipid staining of 2D- and 3D-cultured 3T3-L1 cells with BODIPY, DAPI, and phalloidin are shown in upper panels (scale bar; 100 μm). In the lower panels, 2D- and 3D-cultured 3T3-L1 cells at Day 7 were subjected to qPCR analysis of *Ppaγ*, *Cebpa*, *Ap2* (*Fabp4*), *AdipoQ*, and *leptin*, and the resulting plots of *Pparγ*, *Cebpa,* and *Ap2* are shown *(AdipoQ* and *leptin* expressions were not detected in both 2D and 3D). All experiments were performed in duplicate using fresh preparations consisting of 5 or 16 of 3D spheroids each for lipid staining or qPCR analysis, respectively, or 2D-cultured cells (*n* = 4 wells from 6 well culture dish) for both analyses. Data are presented as arithmetic mean ± standard error of the mean (SEM). ** *p* < 0.01 (unpaired *t*-test).

In our previous studies, we found a significant diversity between 2D- and 3D-cultured 3T3-L1 cells including trypsin sensitivity and efficiency of adipogenesis [8,9,10,11]. Based upon these collective findings, we hypothesized that spontaneous adipogenesis may have occurred in the 3D spheroid form of the 3T3-L1 cells. To examine this possibility further, we performed lipid staining with BODIY and the gene expression of adipogenesis-related factors, including *peroxisome proliferator-activated receptor γ* (*Pparγ*), *CCAAT/enhancer-binding protein alpha* (*Cebpa*), *Ap2* (*fatty acid-binding protein 4*; *Fabp4*), *AdipoQ* (*adiponectin*), and *leptin.* As shown in Figure 1, positive staining with BODIPY, and a significant upregulation of the expression of several adipogenesis-related genes including *Pparγ*, *Cebpa*, and *Ap2* were observed in the 3D 3T3-L1 spheroid (*AdipoQ* and *leptin* expressions were not detected in both 2D and 3D).

### 3.2. Differentially Expressed Genes in 2D- and 3D-Cultured 3T3-L1 Cells

**Figure 2:**
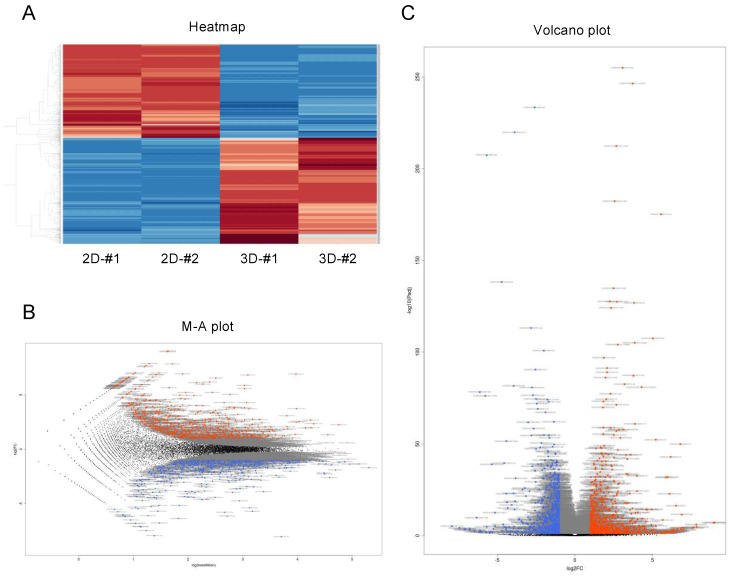
Differentially expressed genes (DEGs) between 2D- and 3D-cultured 3T3-L1 cells. Genes that are expressed differentially between 2D- and 3D-cultured 3T3-L1 cells were demonstrated by a hierarchical clustering heatmap (**A**), an M–A plot (**B**), and a volcano plot (**C**). M–A plot represents relationship between mean expression values (log (base mean); *x* axis) and magnitude of gene expression change (log2 of fold change; *y* axis), and volcano plot represents the relationship between the magnitude of gene expression change (log2 of fold change; *x* axis) and statistical significance of this change (−log10 of false discovery rate (FDR); *y* axis). Colored points represent differentially expressed genes (cutoff FDR < 0.05) and/or the magnitude of change ≥2 that are either overexpressed (red) or underexpressed (blue) in 2D-cultured, compared with 3D-cultured 3T3-L1 cells.

To elucidate the unidentified and underlying mechanism for causing this biological diversity between 2D- and 3D-cultured 3T3-L1 cells, we compared the gene expression profiles, and differentially expressed genes (DEGs) were detected. We found 1551 DEG genes with a significance level of <0.05 (FDR) and absolute fold change ≥2 (Figure 2). Among these, 826 genes were found to be significantly upregulated, and 725 genes were significantly downregulated. The list of upregulated or downregulated top 60 genes is shown in Supplementary Table S2.

### 3.3. Ingenuity Pathway Analysis (IPA) and GO Enrichment Analysis for DEGs

**Figure 3:**
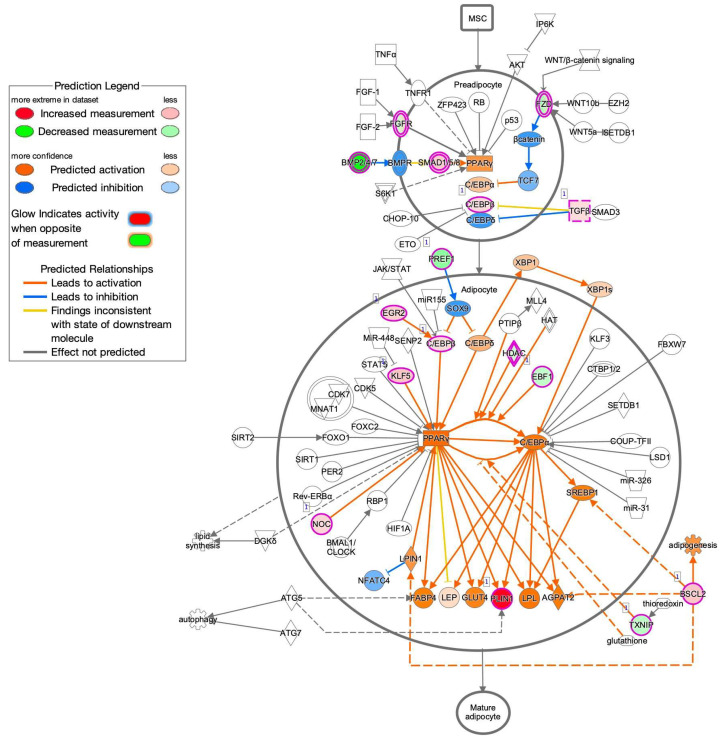
Possible networks of adipogenesis-related signaling during the maturation of adipocytes obtained by the IPA analysis database.

**Figure 4:**
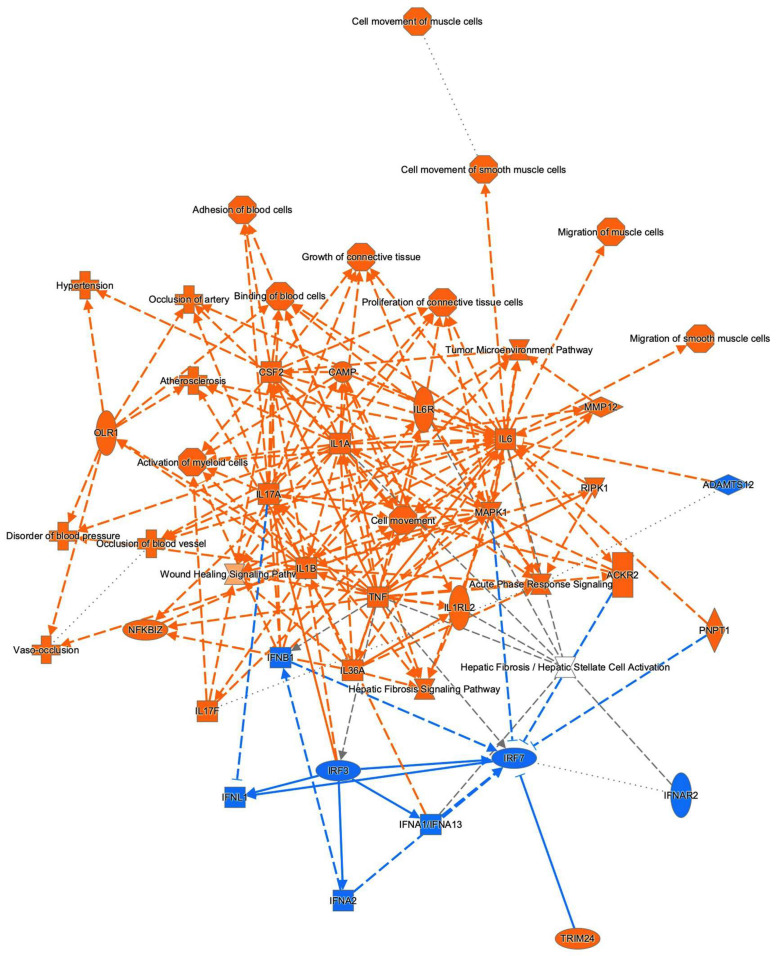
The graphical summary of the biological process network of DEGs. Prediction legend is shown in Figure S1.

**Figure 5:**
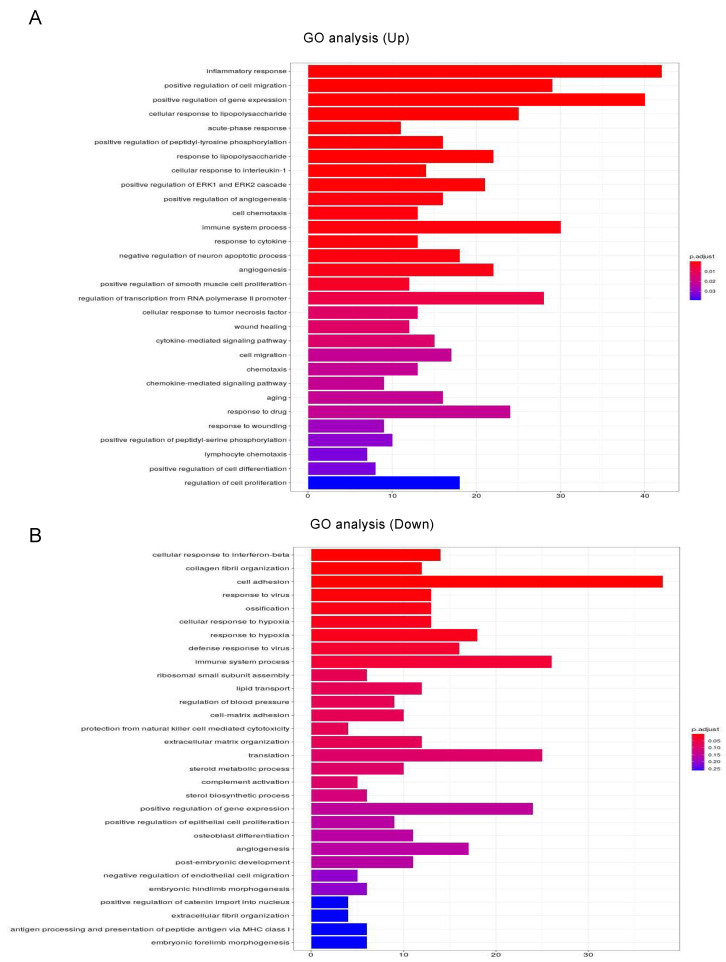
GO enrichment analysis and Ingenuity Pathway Analysis (IPA) of DEGs in 2D- and 3D-cultured 3T3-L1 cells. Upregulated (**A**) and downregulated (**B**) mRNA-enriched biological functions were validated by GO analysis.

To estimate the possible functional properties of the DEGs, Ingenuity Pathway Analysis (IPA) and GO enrichment analysis (QIAGEN, Redwood City, CA, USA) were performed. For example, an IPA analysis database could readily demonstrate between possible networks of adipogenesis-related signaling during the maturation of adipocytes, as shown in Figure 3. A graphical summary of an estimated network of the biological processes based upon the IPA and GO enrichment analyses of the upregulated or downregulated DEGs is shown in Figure 4. Among these, lists of the top 30 upregulated or downregulated GO terms are shown in Figure 5A,B, respectively. The most notable changes that were observed included the upregulation of inflammatory response and the regulation of cell migration and gene expression and the downregulation of cell adhesion.

**Figure 6:**
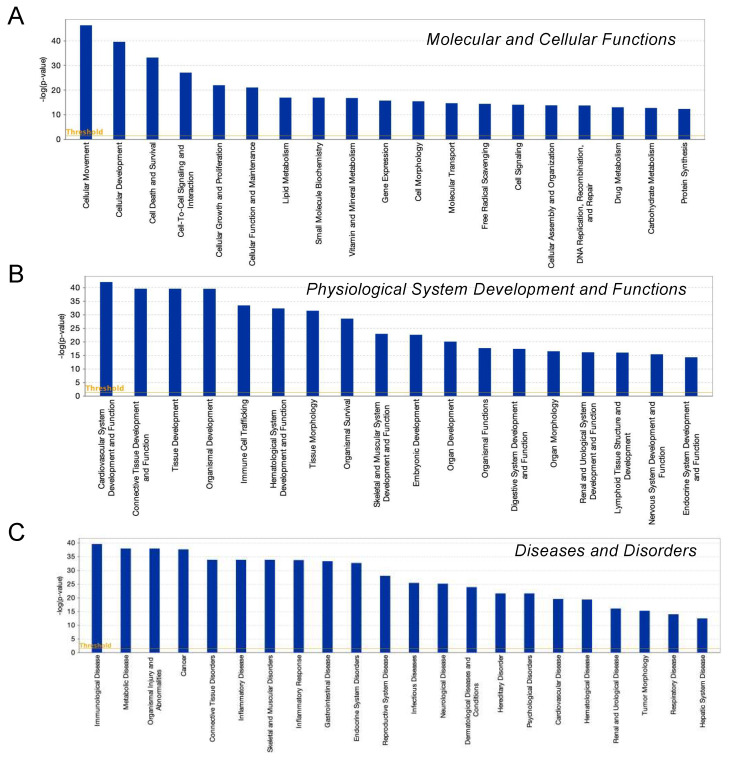
IPA analysis of DEGs. Overlapping canonical 25 upregulated pathways were estimated by the IPA analysis. Among three biophysiological categories, molecular and cellular functions (**A**), physiological system development and function (**B**), and diseases and disorders (**C**), the bars correspond to the top 18–22 canonical pathways that surpassed the Ingenuity statistical threshold using the Benjamini–Hochberg multiple testing correction of Fisher’s exact test.

**Figure 7:**
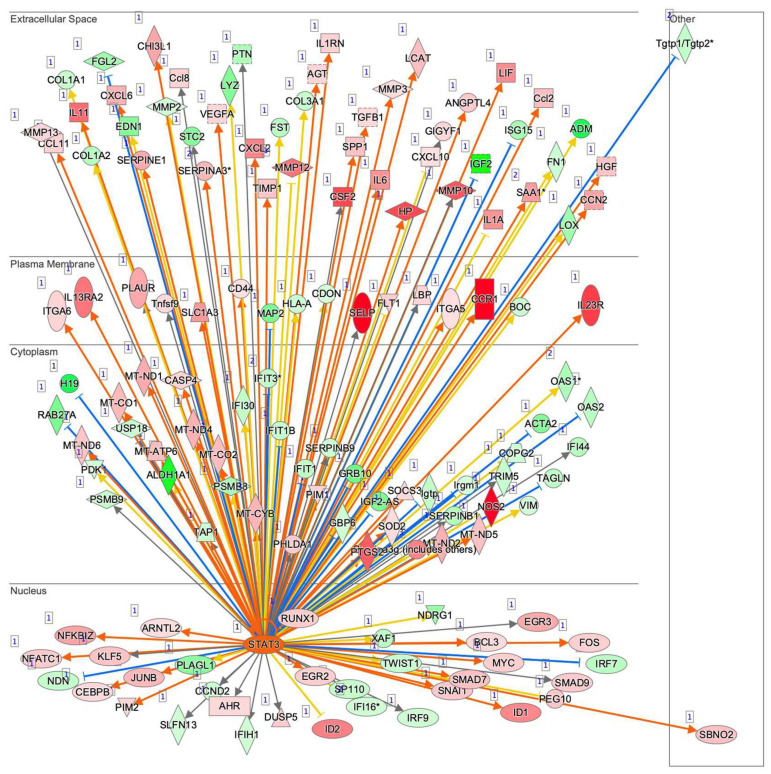
Upstream regulator analysis by IPA. Upstream analysis in IPA identified TGFβ1, STAT3, IL6, AGT, FOS, and MYC as master regulators. The graphs and the networks of each regulator were obtained through the use of IPA (QIAGEN Inc., https://www.qiagenbioinformatics.com/products/ingenuity-pathway-analysis, accessed on 3 November 2021). Among these, the STAT3 network is demonstrated, and others are shown in Figure S2. Prediction legend is shown in Figure S1.

**Figure 8:**
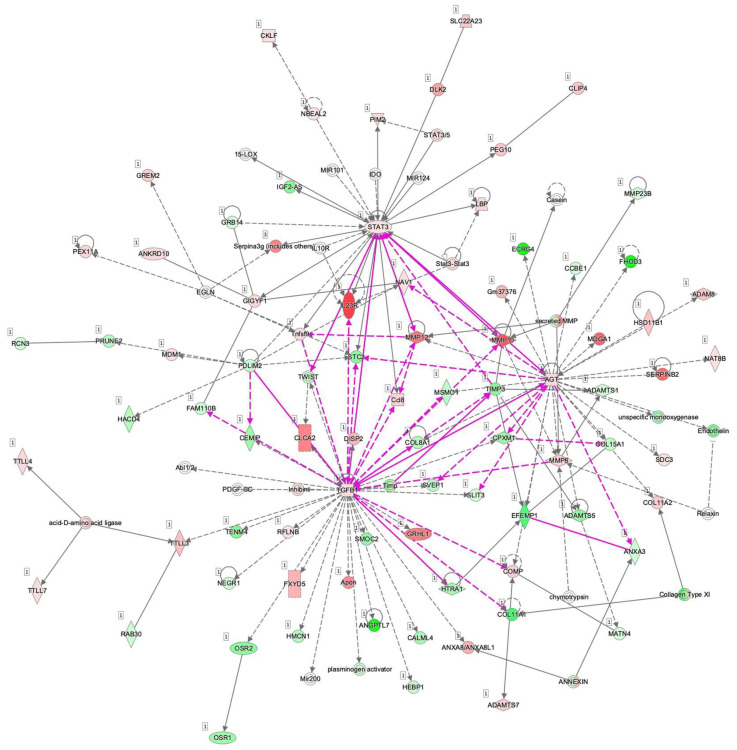
Merged causal networks related to AGT (network 5), STAT3 (network 7), and TGFβ1 (network 10). Prediction legend is shown in Figure S1.

To examine this issue further, a canonical pathway analysis using IPA was performed to estimate possible DEG functions related to several already known biological pathways. Bar graphs of Figure 6A–C revealed the estimated pathway most likely related (*p* < 0.05) with the following three categories: molecular and cellular functions (A), physiological system development and functions (B), and diseases and disorders (C). Upstream analysis of IPA allowed 50 possible regulators to be identified, and among these, 6 regulators—transforming growth factor β1 (TGFβ1), signal transducer and activator of transcription 3 (STAT3), interleukin 6 (IL6), angiotensinogen (AGT), FOS, and MYC—were, in fact, significantly upregulated (Table S3), suggesting these were major upstream regulators involved in the diversity between 2D- and 3D-cultured 3T3-L1 cells. Causal networks of these regulators indicated that STAT3 (Figure 7) and FOS (Figure S2) were estimated to be directly connected with TGFβ1, IL6, AGT, and MYC (Figure S2). To further estimate the possible roles of these 6 regulators within pathophysiological functions, the predicted top 14 diseases and functions networks, for which the IPA network score was more than 30 (Table 1 and Figure S3A,B) were evaluated. Rather interestingly, AGT, STAT3, and TGFβ1 were all found to be critical upstream regulators of networks 5, 7, and 10, and those regulators were linked with each other (Figure 8 and Figure S4). These collective observations suggest that STAT3 may be the master upstream regulator for causing these unique biological properties of the above 3D 3T3-L1 spheroids.

## 4. Discussion

To characterize the adipogenic differentiation of 3T3-L1 cells, their gene expression profiles were extensively investigated [19,20]. Most previous studies have used 2D-cultured 3T3-L1 cells grown by conventional culture methods using standard culture dishes and culture medium. In contrast, only a few studies have appeared in which different culture methods have been used, including Matrigel^®^ assisted 2D cultures and 3D spheroid cultures. Interestingly, Josan et al. recently reported that lipid accumulation and the gene expression of 3T3-L1 cells that had been cultured and differentiated on a 250 μm layer of Matrigel^®^ were relatively different from comparable cells that were cultured on tissue culture-coated polystyrene surfaces; that is, Matrigel^®^ appeared to cause a significantly enhanced lipid accumulation, as well as the expression of transcription factors and markers involved in lipogenesis and adipocyte maturity, and induce a significant downregulation of ECM markers. Therefore, based on these observations, they hypothesized that the differentiation of 3T3-L1 cells on Matrigel^®^ may be closer to the differentiation that occurred under in vivo conditions [21]. Our recent studies and the current study using the 3D 3T3-L1spheroid cultures also led to similar observations of a different level of efficiency of adipogenesis and the expressions of adipogenesis-related factors and several ECM proteins [8,9,10,11,12].

In terms of the underlying mechanisms responsible for causing such different biological properties between 2D- and 3D-cultured 3T3-L1, Lee et al. recently reported on a global quantitative proteomic profiling analysis of three 3D-cultured 3T3-L1 cells groups—namely, preadipocytes, adipocytes, and co-cultured adipocytes with macrophages, and a comparison was made with the 2D-cultured counterparts using 2D-nanoLC-ESI-MS/MS with iTRAQ labeling [22]. Among a total of 2885 shared proteins from 6 types of adipose cells, they found that 48 proteins involved in carbohydrate metabolism (e.g., PDHα, MDH1/2, FH) and the mitochondrial fatty acid beta-oxidation pathway (e.g., VLCAD, ACADM, ECHDC1, ALDH6A1) were relatively upregulated in the 3D co-culture model, compared with their corresponding counterparts in 2D and 3D mono-cultured cells, and 12 proteins implicated in the cellular component organization (e.g., ANXA1, ANXA2) and the cell cycle (e.g., MCM family proteins) were downregulated. Alternatively, in a study of the underlying genomic mechanisms responsible for the different phenotypes, in which 2D monolayers and 3D spheroid 3T3-L1 adipocyte cultures were subjected to transcriptome analysis, Turner et al. reported a significant differential expression of genes related to adipogenesis, including adipocytokine signaling, fatty acid metabolism, and PPAR-γ signaling [23]. They also showed the downregulation of matrix metalloproteinases (MMPs), integrin, actin–cytoskeleton-associated genes, and the downregulation of Rho/GTPase3 expression in 3D spheroids, compared with 2D monolayers, indicating suppression of the Rho-ROCK pathway and thereby promoting adipogenic differentiation [23]. In the current study, we also performed transcriptome analysis using an IPA upstream analysis of 2D- and 3D-cultured 3T3-L1 preadipocytes and found that six upstream regulators—TGFβ1, STAT3, IL6, AGT, FOS, and MYC—appear to be pivotally involved in the possible mechanisms responsible for inducing a diversity of biological properties between the two systems. In addition, based upon further estimations of the predicted top 14 diseases and functions networks, we conclude that STAT3 appears to be the most critical upstream regulator among the potential regulators.

The STAT family of proteins consists of seven related transcription factors comprising STAT1 to STAT6 [24]. Among these, under basal conditions, a latent form of STAT3 remains in the cytoplasm and changes into the transcriptionally active form in response to external signals, including several cytokines such as IL6, IL10, the leukemia inhibitory factor (LIF), the ciliary neurotrophic factor (CNTF), growth factors such as TGFβ1, platelet-derived growth factor (PDGF), the epidermal growth factor (EGF), etc. [25,26,27]. Interestingly, in the current study, we also observed significant upregulations of IL6 and TGFβ1, in addition to STAT3 in an IPA upstream regulator analysis (Supplementary Table S3). It was also reported that IL6 enhances the expression of AGT, which was also found to be upregulated in the current analysis, via the STAT3 activation in hepatocytes [28,29,30]. Several lines of evidence suggest that STAT3 plays a functional, pivotal role in modulating the biology of cancer cells, by affecting their energy metabolism and the metabolism of glucose and lipids [31,32,33,34]. In addition, STAT proteins are also known to be involved in adipogenesis and the associated process of lipogenesis that proceeds through canonical activation through phosphorylation, nuclear translocation, and gene activation [35,36,37,38]. In fact, it has been reported that the JAK2/STAT3 pathway regulates the early stage of adipogenesis through regulating C/EBPβ transcription [37], and IL-6-STAT3 signaling has also been identified as a strong inducer of adipocyte browning [39,40]. These collective observations provide rational support for our speculation that upregulated STAT3 in the 3D spheroid form of the 3T3-L1 cells may lead to spontaneous adipogenesis. More interestingly, previous studies indicated that STAT3 is also involved in gravity-induced biological effects [41,42,43]. Since it is believed that gravity-induced effects may be one of the significant differences between 2D and 3D culture systems, we reasonably speculated that gravity changes toward 3T3-L1 cells during 3D spheroid culturing may affect the STAT3-related network resulting in several different biological aspects, compared with their 2D culture, as described above. However, at the time of this writing, this issue remains to be unidentified. Based upon our current study, we speculated that the 3D spheroid form may be closer to in vivo conditions such as an organ, compared with 2D-cultured cells simply on a culture plate. However, at present, it is difficult to conclude that 3D cultures are really closer to in vivo conditions, compared with 2D cultures, because of insufficient experimental observations. Therefore, our future research will include additional characterizations of the 2D- and 3D-cultured 3T3-L1 cells between before and after adipogenesis, comparisons with in vivo derived cells, and further studies using knockdown STAT3 and other possible regulators using several other sources of cells in addition to 3T3-L1 cells.

## 5. Conclusions

The findings presented herein suggest that STAT3 has a critical role in regulating the unique biological properties of 3D spheroids that are produced from 3T3-L1 preadipocytes.

## Figures and Tables

**Table 1:** Lists of the top 14 causal networks of diseases and functions with IPA network score of more than 30.

No.	Top Diseases and Functions	Score	Focus Molecules
1	Cell Signaling, Dermatological Diseases and Conditions, Immunological Disease	48	34
2	Cancer, Gastrointestinal Disease, Organismal Injury and Abnormalities	41	31
3	Neurological Disease, Protein Synthesis, RNA Damage and Repair	41	31
4	Drug Metabolism, Molecular Transport, Nucleic Acid Metabolism	34	28
5	Connective Tissue Disorders, Organismal Injury, and Abnormalities, Skeletal and Muscular Disorders	34	28
6	Cell Death and Survival, Cellular Development, Connective Tissue Development and Function	34	28
7	Gastrointestinal Disease, Inflammatory Disease, Inflammatory Response	32	27
8	Cell Signaling, Nervous System Development and Function, Post-Translational Modification	32	27
9	Hematological Disease, Hereditary Disorder, Metabolic Disease	30	26
10	Embryonic Development, Organismal Injury, and Abnormalities, Renal and Urological Disease	30	26
11	Endocrine System Disorders, Gastrointestinal Disease, Immunological Disease	30	26
12	Dermatological Diseases and Conditions, Hair and Skin Development and Function, Organismal Injury and Abnormalities	30	26
13	Auditory Disease, Cell Morphology, RNA Post-Transcriptional Modification	30	26
14	Cancer, Cell Death, and Survival, Organismal Injury, and Abnormalities	30	26

## Data Availability

The data presented in this study are available on request from the corresponding author. The data are not publicly available due to the regulation policy of use of IPA (QIAGEN Inc., https://www.qiagenbioinformatics.com/products/ingenuity-pathway-analysis, accessed on 3 November 2021).

## Supplementary Materials

The following supporting information can be downloaded at: https://www.mdpi.com/article/10.3390/cells11020300/s1. Figure S1: Prediction Legend. Figure S2: Upstream regulator analysis by IPA. Upstream analysis in IPA identified TGFβ1, STAT3, IL6, AGT, FOS, and MYC as major regulators. The graphs and the networks of each regulator were obtained through the use of IPA (QIAGEN Inc., https://www.qiagenbioinformatics.com/products/ingenuity-pathway-analysis, accessed on 3 November 2021). Among these, the STAT3 network is shown in Figure 7, and others are shown here. The prediction legend is shown in Figure S1. Figure S3: Top 14 causal networks of diseases and functions with IPA network score more than 30 (A: networks 1, 2, 3, 4, 6 and 8, B: networks 9, 11, 12, 13, and 14). Prediction legend is shown in Figure S1. Figure S4: Causal networks related to AGT (network 5, A), STAT3 (network 7, B), and TGFβ1 (network 10, C). The prediction legend is shown in Figure S1. Table S1: Primers for qPCR. Table S2: Top 60 genes of upregulation and downregulation. Table S3: Possible upstream regulators by IPA upstream analysis.
