# Supplementary material for: STAT3 Is the Master Regulator for the Forming of 3D Spheroids of 3T3-L1 Preadipocytes
Source: Cells. 2022 Jan 16;11(2):300. doi: 10.3390/cells11020300 (PMC8774605; doi:10.3390/cells11020300)
Supplement: Supplementary file 1 [file cells-11-00300-s001.zip › cells-1505616-supplementary.pdf]

Table S1

Supplementary Table 1. Primers for qPCR.

Gene	Forward primer (5' to 3')	Reverse primer (5' to 3')	TaqMan probe (5' to 3')
Pparγ	CTGCTCCACACTATGAAGACAT	TGCAGGTTCTACTTTGATCGC	/FAM/AGCTGACCC/ZEN/AATGGTTGCTGATTACA/IABkFQ/
Cebpα	ACAAGAACAGCAACGAGTACC	TCATTGTCACTGGTCAACTCC	/FAM/CGCAAGAGC/ZEN/CGAGATAAAGCCAAAC/IABkFQ/
Ap2	AAATCACCGCAGACGACAG	CCTTTCATAACACATTCCACCAC	/FAM/TGAAGAGCA/ZEN/TCATAACCCTAGATGGCG/IABkFQ/
Leptin	GTGCCTATCCAGAAAGTCCAG	AATGAAGTCCAAGCCAGTGA	/FAM/ACCGACTGC/ZEN/GTGTGTGAAATGTCA/IABkFQ/
AdipoQ	GCAGGATTAAGAGGAACAGGAG	TGTCTGTACGATTGTCAGTGG	/56-FAM/ACGACACCA/ZEN/AAAGGGCTCAGGAT/3IABkFQ/
36b4	TTATAACCCTGAAGTGCTCGAC	CGCTTGTACCCATTGATGATG	/FAM/AGGCCCTGC/ZEN/ACTCTCGCTT/IABkFQ/

Table S2

Supplementary Table 2. Top 60 genes of upregulation and down regulation

	Upregulation (Total: 826 genes)			Downregulation (Total: 725 genes)		
	log 2 Fold Change (3D/2D)	Gene symbol	Gene biotype	log 2 Fold Change (3D/2D)	Gene symbol	Gene biotype
1	9.00	Selp	protein_coding	-8.01	Ogn	protein_coding
2	8.94	U90926	protein_coding	-7.93	Tinag	protein_coding
3	7.85	Ranbp3l	protein_coding	-7.44	Inmt	protein_coding
4	7.82	Plin1	protein_coding	-7.12	Pgm5	protein_coding
5	7.66	Ccr1	protein_coding	-7.06	Rnf112	protein_coding
6	7.56	Rab15	protein_coding	-6.79	1500015O10Rik	protein_coding
7	7.00	Gm16548	lincRNA	-6.78	Omd	protein_coding
8	6.98	Gm567	lincRNA	-6.76	Angptl7	protein_coding
9	6.89	Lgr6	protein_coding	-6.48	Bmp4	protein_coding
10	6.89	Gucy2g	protein_coding	-6.34	Fhod3	protein_coding
11	6.89	Saa3	protein_coding	-6.17	Cpa1	protein_coding
12	6.83	Ing4	protein_coding	-6.14	9130208D14Rik	unprocessed_pseudogene
13	6.83	Nos2	protein_coding	-6.13	Itgbl1	protein_coding
14	6.80	Tnn	protein_coding	-6.08	Trhde	protein_coding
15	6.63	Accsl	protein_coding	-6.04	Tek	protein_coding
16	6.60	Camp	protein_coding	-6.01	Fgf14	protein_coding
17	6.58	Gm43609	TEC	-5.77	Ifi27l2a	protein_coding
18	6.52	Hr	protein_coding	-5.73	Slc4a10	protein_coding
19	6.49	Myo1g	protein_coding	-5.69	Igf2	protein_coding
20	6.49	Rasgrp2	protein_coding	-5.67	Aldh1a7	protein_coding
21	6.44	Stfa3	protein_coding	-5.5	Optc	protein_coding
22	6.43	Snord43	miRNA	-5.45	Nkain2	protein_coding
23	6.36	Gm37309	TEC	-5.36	Clec3b	protein_coding
24	6.32	Cd79a	protein_coding	-5.32	Rgag1	protein_coding
25	6.32	Chad	protein_coding	-5.25	Ctsc	protein_coding
26	6.26	Nppb	protein_coding	-5.15	Aldh1a1	protein_coding
27	6.26	Gm14508	antisense	-5.15	Sult1a1	protein_coding
28	6.25	Ngef	protein_coding	-4.72	Chrdl1	protein_coding
29	6.2	Cyth4	protein_coding	-4.69	Ccl6	protein_coding
30	6.18	Cck	protein_coding	-4.62	Agtr2	protein_coding
31	6.14	Gm16587	antisense	-4.56	Fmod	protein_coding
32	6.13	2700080J24Rik	TEC	-4.55	Krt8	protein_coding
33	6.11	Slco4a1	protein_coding	-4.43	Gm42922	TEC
34	6.07	Notumos	antisense	-4.43	Prex2	protein_coding
35	6.07	Spink6	protein_coding	-4.41	Nrk	protein_coding
36	5.98	Ces1f	protein_coding	-4.41	Prokr1	protein_coding
37	5.93	Kcnk10	protein_coding	-4.41	Me3	protein_coding
38	5.93	Gm26621	processed_transcript	-4.37	Adm	protein_coding
39	5.93	Lce1f	protein_coding	-4.36	Actr3b	protein_coding
40	5.93	Mir155hg	lincRNA	-4.33	H19	lincRNA
41	5.93	Gm26514	lincRNA	-4.3	Dchs2	protein_coding
42	5.92	Hsbp1l1	protein_coding	-4.29	Gm27786	misc_RNA
43	5.86	Lcn2	protein_coding	-4.25	Bnc1	protein_coding
44	5.85	RP23-14P17.5	sense_intronic	-4.16	Gpihbp1	protein_coding
45	5.85	Il23r	protein_coding	-4.15	Cpz	protein_coding
46	5.85	Ucn2	protein_coding	-4.15	Krt7	protein_coding
47	5.85	Scg5	protein_coding	-4.07	Sorbs2	protein_coding
48	5.85	Gm26789	lincRNA	-4.01	Mylk3	protein_coding
49	5.85	2310007B03Rik	protein_coding	-3.96	Gm16559	antisense
50	5.84	Lrg1	protein_coding	-3.96	Myl9	protein_coding
51	5.84	Rdh8	protein_coding	-3.95	Sdpr	protein_coding
52	5.77	Greb1	protein_coding	-3.91	Mlana	protein_coding
53	5.77	Atp13a4	protein_coding	-3.88	Col11a1	protein_coding
54	5.77	Vipr2	protein_coding	-3.88	Adh1	protein_coding
55	5.77	Rnf183	protein_coding	-3.86	Mfap5	protein_coding
56	5.77	Gm37755	TEC	-3.84	Gm27483	misc_RNA
57	5.76	Pla2g4b	protein_coding	-3.82	Cdh26	protein_coding
58	5.76	Gm43702	TEC	-3.81	Sema5b	protein_coding
59	5.67	Mroh4	protein_coding	-3.76	Gm14434	protein_coding
60	5.67	Snora34	snoRNA	-3.7	Efemp1	protein_coding

Table S3

Supplementary Table 3. Possible upstream regulators by IPA upstream analysis

Upstream Regulator	Exp Fold Change	Molecule Type	Predicted Activation State	Activation z-score	p-value of overlap
TNF		cytokine	Activated	4.882	2.53E-55
lipopolysaccharide		chemical drug	Activated	3.541	5.27E-54
PDGF BB		complex	Activated	5.740	1.95E-50
TGF 1	↑ 1.143	growth factor		1.960	1.61E-48
STAT3	↑ 1.029	transcription regulator	Activated	4.465	1.83E-47
dexamethasone		chemical drug	Inhibited	-3.535	4.10E-46
AGT	↑ 1.301	growth factor		1.722	2.87E-44
beta-estradiol		chemical-endogenous	Activated	2.178	4.27E-43
IFNG		cytokine		1.452	2.24E-42
IL1		cytokine	Activated	4.239	7.93E-41
KRAS		enzyme	Activated	3.919	5.68E-36
tretinoin		chemical-endogenous		0.701	1.32E-35
CG		complex		0.728	2.23E-35
IL6	↑ 3.458	cytokine	Activated	4.804	7.10E-35
NFKBIA		transcription regulator		1.145	1.92E-31
HRAS		enzyme	Activated	3.153	2.51E-31
TP53		transcription regulator		-1.913	3.26E-31
JUN		transcription regulator	Activated	3.603	1.04E-29
CTNNB1		transcription regulator		0.348	1.83E-29
U0126		chemical drug	Inhibited	-3.583	2.20E-28
IL10RA		transmembrane receptor		-1.573	4.15E-28
poly rI:rC-RNA		biologic drug		0.805	4.20E-28
FOS	↑ 1.512	transcription regulator	Activated	3.086	5.01E-28
SB203580		chemical drug	Inhibited	-3.908	2.08E-27
TAZ		enzyme		-0.765	2.57E-27
Tgf		group		1.004	3.07E-27
IGF1		growth factor	Activated	4.333	4.89E-27
IKBKB		kinase		1.505	4.90E-27
YAP1		transcription regulator		-0.181	1.66E-26
WNT3A		cytokine		0.531	2.02E-26
OSM		cytokine	Activated	3.136	3.80E-26
G protein		group		-1.722	3.87E-26
ESR2		ligand-dependent nuclear receptor	Activated	2.027	4.41E-26
CHUK		kinase		1.880	1.19E-25
KDM1A		enzyme		0.895	2.69E-25
MYC	↑ 1.548	transcription regulator		0.041	2.95E-25
GNA15		enzyme			8.09E-25
PPARGC1A		transcription regulator	Activated	2.038	1.76E-24
PNPT1		enzyme	Activated	4.518	1.98E-24
cardiotoxin		chemical-other	Activated	2.268	2.42E-24
CITED2		transcription regulator		0.676	2.54E-24
IL17A		cytokine	Activated	4.552	2.63E-24
Alpha catenin		group		-0.148	2.70E-24
IFNB1		cytokine	Inhibited	-4.301	3.94E-24
NFκB(complex)		complex	Activated	4.280	4.43E-24
Ige		complex	Activated	4.217	5.64E-24
STAT1		transcription regulator	Inhibited	-2.142	7.53E-24
cycloheximide		chemical reagent		0.803	1.25E-23
IKBKG		kinase		0.450	1.87E-23
LY294002		chemical drug	Inhibited	-2.622	2.14E-23

Figure S1

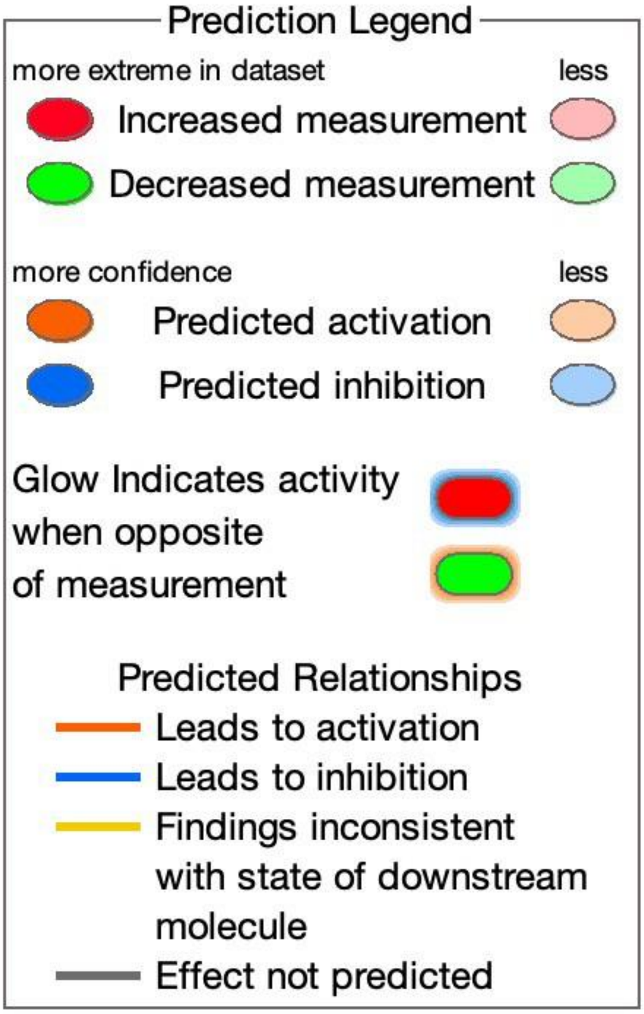

Figure S1: Prediction Legend.

Figure S2

A TGFB1

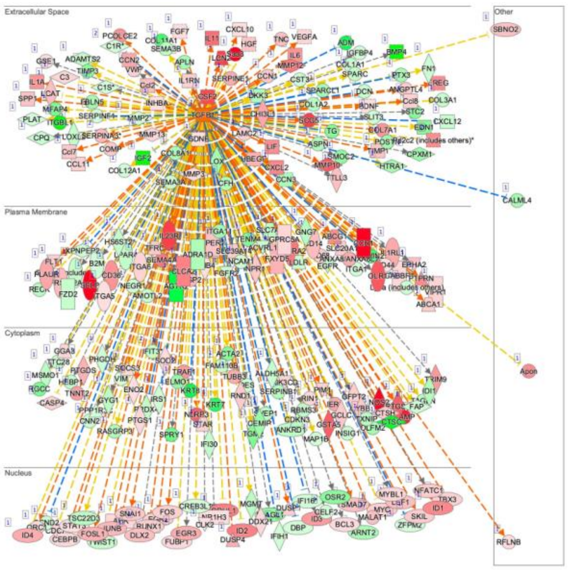

B AGT

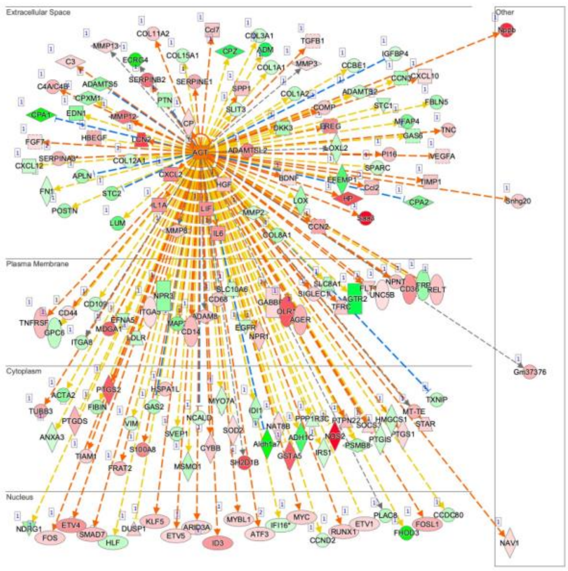

C IL6

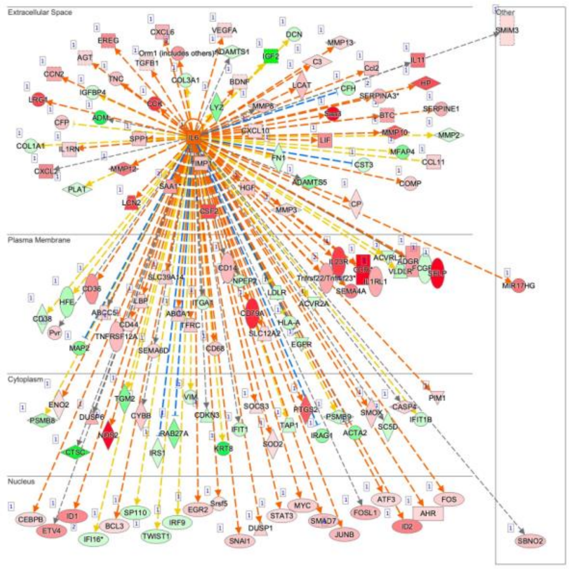

D MYC

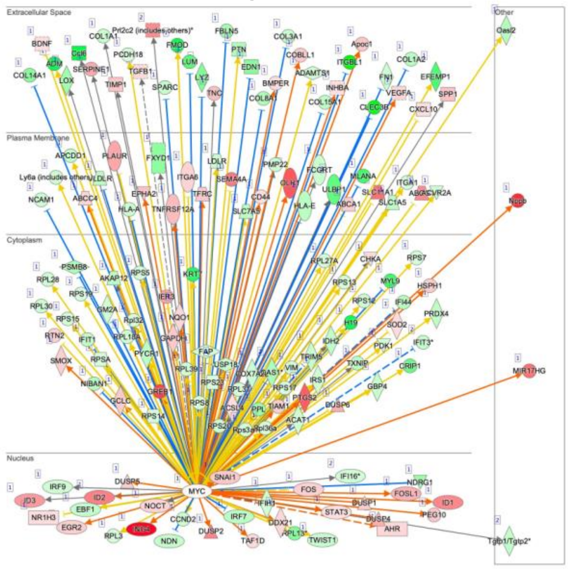

E FOS

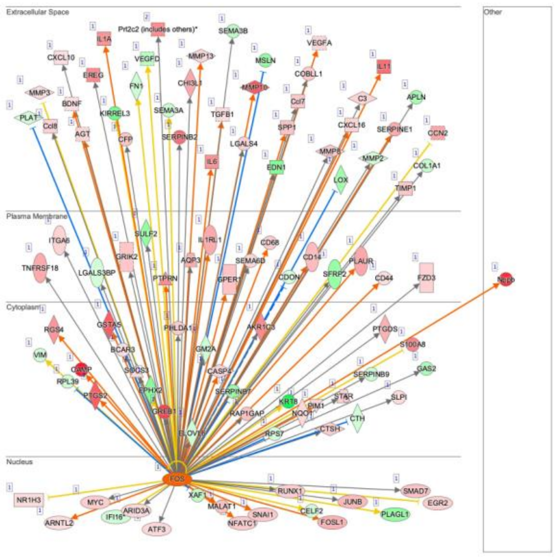

Figure S2: Upstream regulator analysis by IPA. Upstream analysis in IPA identified TGFβ1, STAT3, IL6, AGT, FOS, and MYC as major regulators. The graphs and the networks of each regulator were obtained through the use of IPA (QIAGEN Inc., https://www.qiagenbioinformatics.com/products/ingenuity-pathway-analysis, accessed on 3 November 2021). Among these, the STAT3 network is shown in Figure 7, and others are shown here. The prediction legend is shown in Figure S1.

.

Figure S3A

Network 1

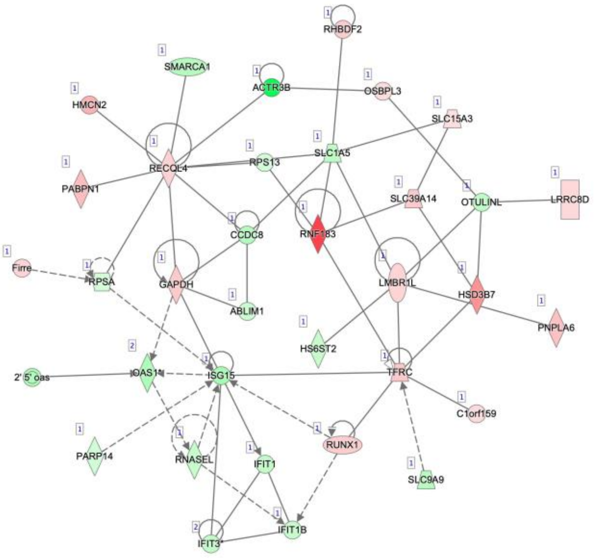

Network 2

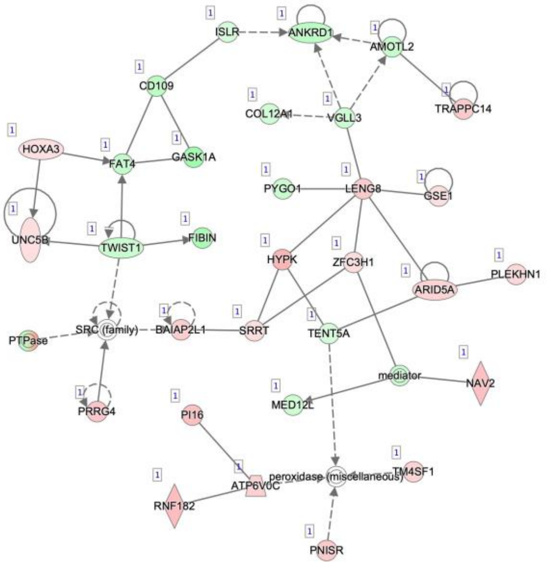

Network 3

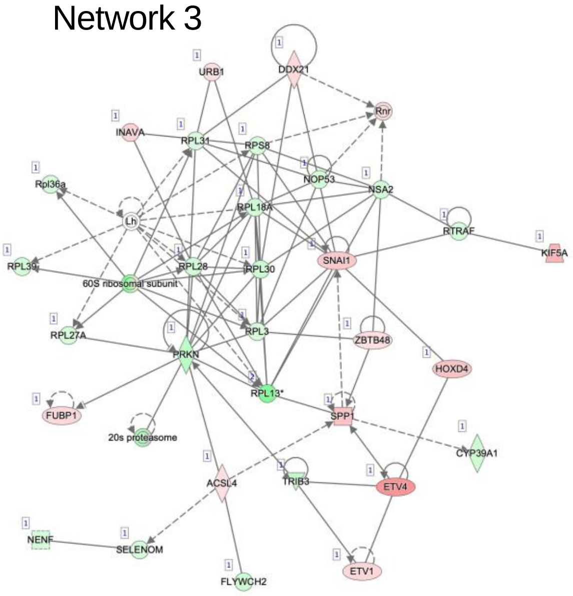

Network 4

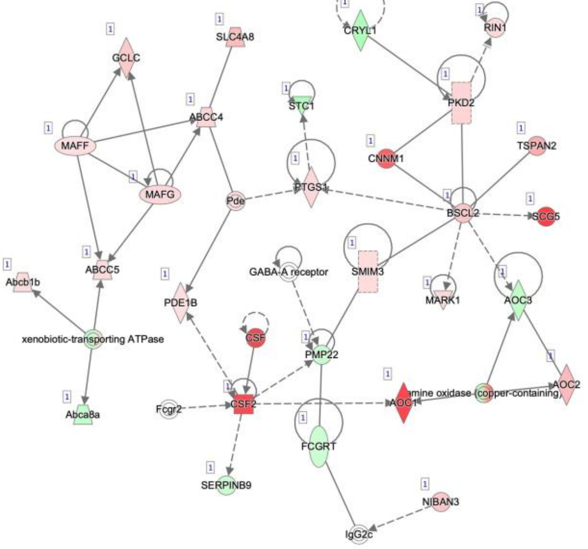

Network 6

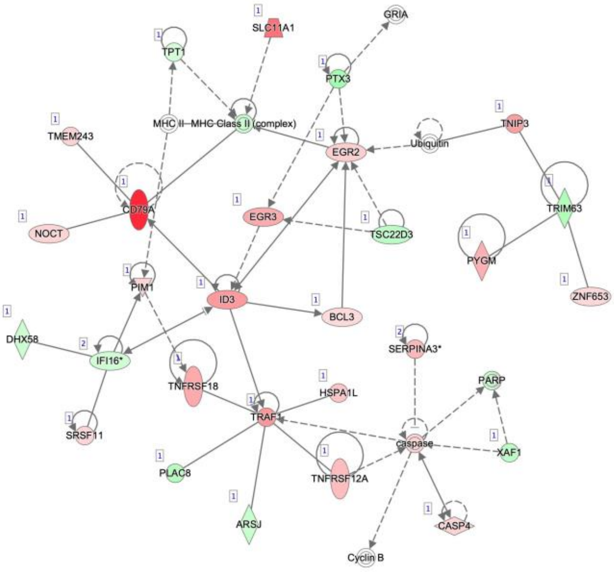

Network 8

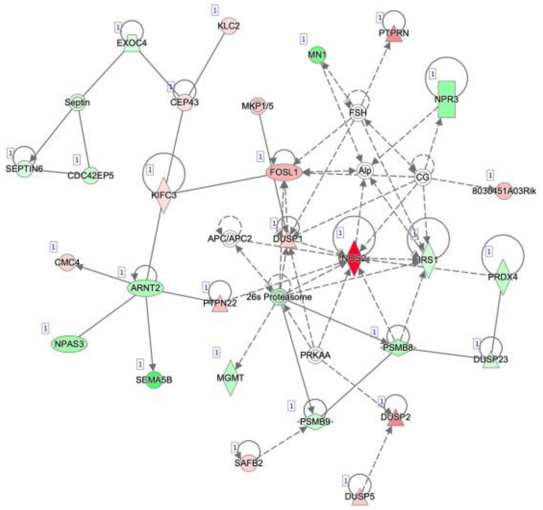

Figure S3B

Network 9

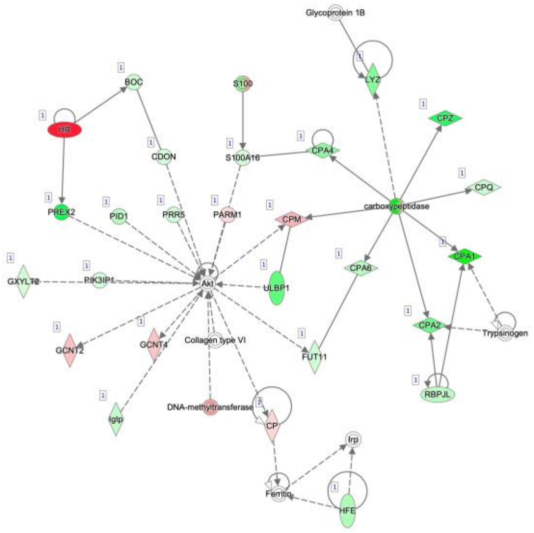

Network 11

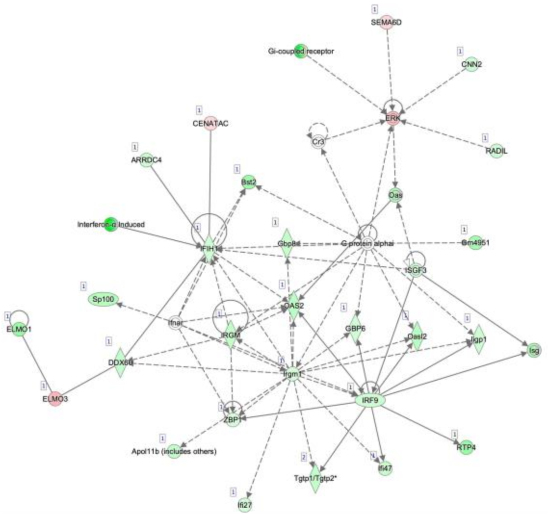

Network 12

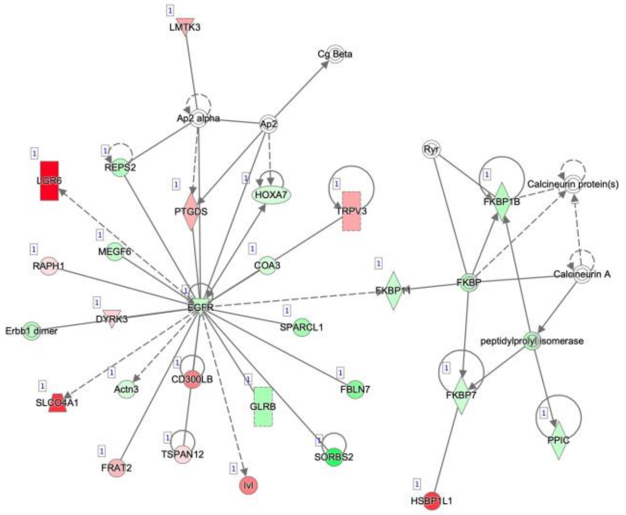

Network 13

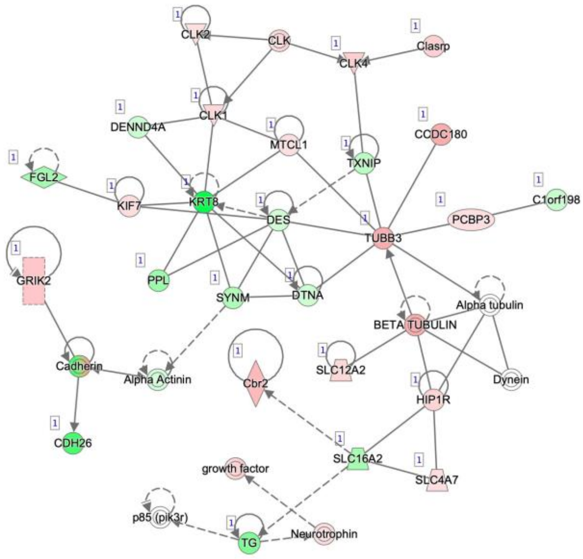

Network 14

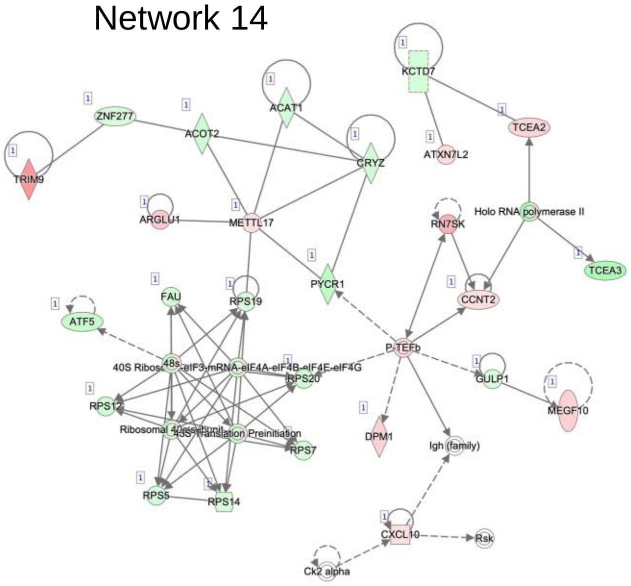

Figure S3: Top 14 causal networks of diseases and functions with IPA network score more than 30 (A: networks 1, 2, 3, 4, 6 and 8, B: networks 9, 11, 12, 13, and 14). Prediction legend is shown in Figure S1.

Figure S4

A

Network 5 (AGT)

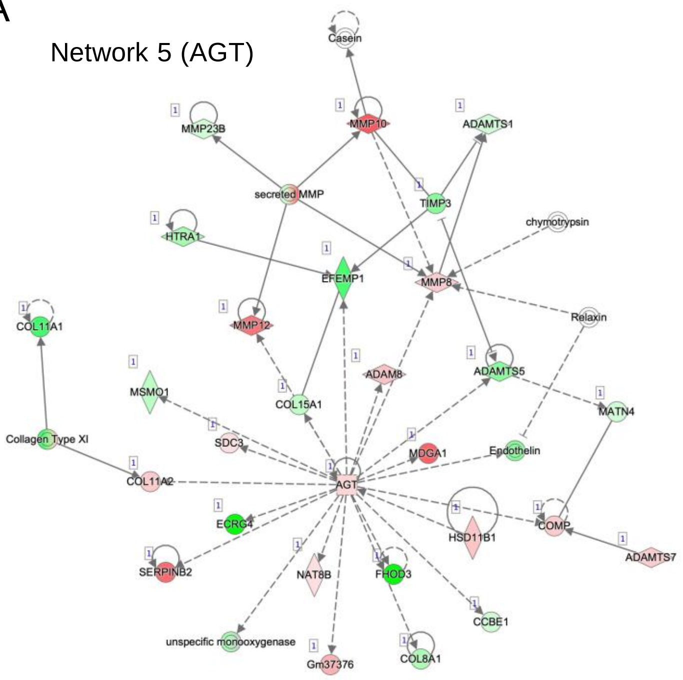

B

Network 7 (STAT3)

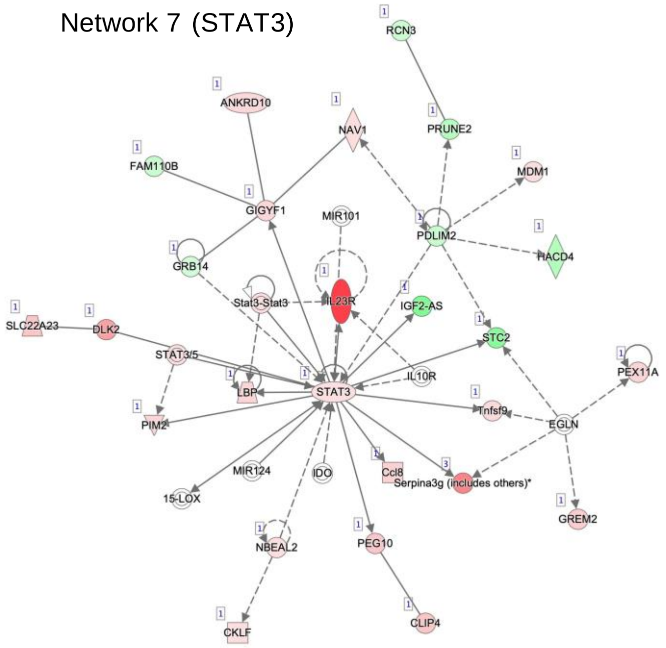

C

Network 10 (TGFB1)

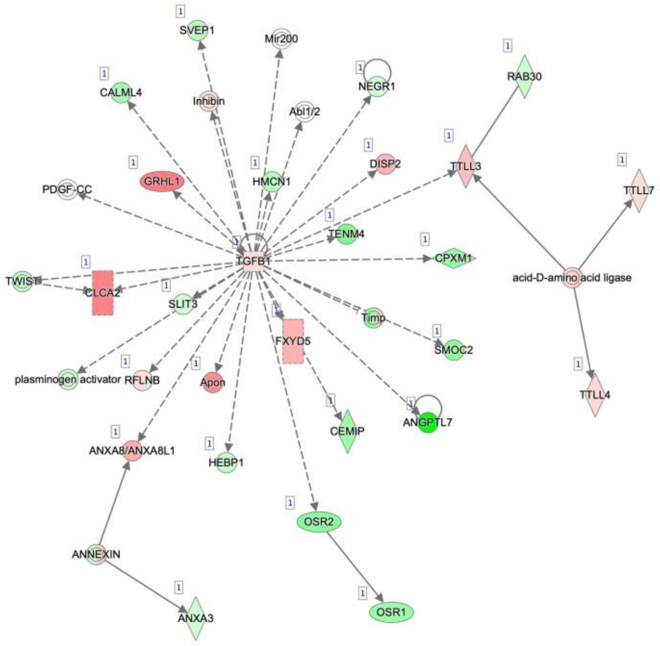

Figure S4: Causal networks related to AGT (network 5, A), STAT3 (network 7, B), and TGFβ1 (network 10, C). The prediction legend is shown in Figure S1.
